# The First Identification of a Narnavirus in Bigyra, a Marine Protist

**DOI:** 10.1264/jsme2.ME22077

**Published:** 2023-03-01

**Authors:** Yuto Chiba, Akinori Yabuki, Yoshihiro Takaki, Takuro Nunoura, Syun-ichi Urayama, Daisuke Hagiwara

**Affiliations:** 1 Laboratory of Fungal Interaction and Molecular Biology (donated by IFO), Department of Life and Environmental Sciences, University of Tsukuba, 1–1–1 Tennodai, Tsukuba, Ibaraki 305–8577, Japan; 2 Research Institute for Global Change (RIGC), Japan Agency for Marine-Earth Science and Technology (JAMSTEC), 2–15 Natsushima-cho, Yokosuka 237–0061, Japan; 3 Super-cutting-edge Grand and Advanced Research (SUGAR) Program, JAMSTEC, 2–15 Natsushima-cho, Yokosuka, Kanagawa 237–0061, Japan; 4 Research Center for Bioscience and Nanoscience (CeBN), JAMSTEC, 2–15 Natsushima-cho, Yokosuka, Kanagawa 237–0061, Japan; 5 Microbiology Research Center for Sustainability (MiCS), University of Tsukuba, 1–1–1 Tennodai, Tsukuba, Ibaraki 305–8577, Japan

**Keywords:** RNA virus, protist, Stramenopiles, *Narnaviridae*, marine virus

## Abstract

Current information on the diversity and evolution of eukaryotic RNA viruses is biased towards host lineages, such as animals, plants, and fungi. Although protists represent the majority of eukaryotic diversity, our understanding of the protist RNA virosphere is still limited. To reveal untapped RNA viral diversity, we screened RNA viruses from 30 marine protist isolates and identified a novel RNA virus named Haloplacidia narnavirus 1 (HpNV1). A phylogenetic ana­lysis revealed that HpNV1 is a new member of the family *Narnaviridae*. The present study filled a gap in the distribution of narnaviruses and implies their wide distribution in Stramenopiles.

High-throughput sequencing (HTS) technology has expanded our knowledge of RNA virus diversity and evolution (Shi *et al.*, 2016; Dolja and Koonin, 2018; Wolf *et al.*, 2018, 2020; Zayed *et al.*, 2022). Metatranscriptomic ana­lyses of aquatic and soil biospheres have detected numerous novel RNA viruses (Urayama *et al.*, 2018; Starr *et al.*, 2019; Wolf *et al.*, 2020; Chen *et al.*, 2022; Neri *et al.*, 2022; Zayed *et al.*, 2022). Furthermore, HTS-based RNA virus identification from organism samples has been intensively conducted for animals, plants, and fungi (Shi *et al.*, 2016, 2018; Gilbert *et al.*, 2019; Sutela *et al.*, 2020; Kawasaki *et al.*, 2021; Mifsud *et al.*, 2022). The findings obtained have expanded our knowledge of RNA virus diversity; however, the target of HTS ana­lyses of organism samples is biased towards a small fraction of eukaryotic taxa (Cobbin *et al.*, 2021).

Protists are defined as eukaryotes other than animals, land plants, and true fungi (Burki *et al.*, 2020), and some are ecologically and/or economically important. Eukaryotic algae and labyrinthulids play an essential role in the ecosystem as primary producers or decomposers (Field *et al.*, 1998; Raghukumar *et al.*, 2001). Moreover, harmful bloom-forming algae, some parasitic protists, and some oomycetes cause illnesses and death in livestock, fishes, crops, and humans (Derevnina *et al.*, 2016; Mitra and Mawson, 2017; Brown *et al.*, 2019; World Health Organization, 2019). Based on the importance of these protists, several RNA viruses have so far been screened and identified (Nagasaki and Yamaguchi, 1997; Takao *et al.*, 2005; Grybchuk *et al.*, 2018; Charon *et al.*, 2019; Cai *et al.*, 2012; Chiba *et al.*, 2020b; Charon *et al.*, 2021). Despite the vast diversity of protists, the number of studies conducted to date has been limited.

In the present study, to reveal untapped RNA virus diversity in protists, we examined RNA virus genome sequences using HTS from 30 isolates of marine heterotrophic protists belonging to Diplonemea (Euglenozoa), Thecofilosea and Imbrecatea (Cercozoa), and Sagenista and Opalozoa (Bigyra, Stramenopiles) (Table S1 and Fig. S1). To the best of our knowledge, no RNA viruses have been reported in these lineages, except for the Aurantiochytrium single-stranded RNA virus (AuRNAV) identified from an isolate of Sagenista (Takao *et al.*, 2005).

Detailed methods are described in the supplemental material. Briefly, 30 protists were cultured with Hemi medium (Tashyreva *et al.*, 2018) or KLB medium (Yabuki and Tame, 2015) at 20°C, and cells were harvested from cultures by centrifugation at 2,400×*g* for 4‍ ‍min. Taxonomic information on each protist is summarized in Table S1. In the present study, we constructed sequencing libraries from pooled cells and single-strain cells for RNA virus screening and complete RNA virus genome identification, respectively. In screening, 30 strains were pooled into pool-1 and pool-2, as shown in Table S1.

Since double‐stranded RNA (dsRNA) is a marker of RNA virus infection (Morris, 1979), dsRNA was purified from cells, and sequencing libraries were constructed using fragmented and primer-ligated dsRNA sequencing (FLDS) technology as previously described (Urayama *et al.*, 2018; Hirai *et al.*, 2021). The details of this method are described in the supplemental material. Libraries were sequenced using the Illumina NovaSeaq 6000 platform with 150 bp paired-end sequences or the Illumina MiSeq platform with 300 bp paired-end sequences (Illumina). More than 400,000 reads were obtained for each library. Raw sequence reads are available in the Short Read Archive database (DDBJ Accession Nos. DRA014844 and DRA014881).

Raw sequence reads were processed as previously described (Hirai *et al.*, 2021) with a custom Perl script (https://github.com/takakiy/FLDS), and cleaned reads were assembled *de novo* using CLC GENOMICS WORKBENCH version 11.0 (CLC Bio) (Urayama *et al.*, 2016, 2018). To obtain full-length sequences, assembled contigs were manually extended using the assembler and Tablet viewer (Milne *et al.*, 2010). We identified full-length sequences using a previously described method (Urayama *et al.*, 2018). Full-length sequences and contigs were annotated by a BLASTX ana­lysis against the NCBI non-redundant protein database and RNA viral protein sequences reported in recent RNA virome studies (Chen *et al.*, 2022; Neri *et al.*, 2022; Zayed *et al.*, 2022). To identify more distantly related RNA viruses, we performed RNA virus detection using hidden Markov model (HMM) profiles, such as RVDB-prot (Bigot *et al.*, 2019) and NeoRdRp (Sakaguchi *et al.*, 2022).

To identify the host organisms of RNA viruses detected in pooled sequencing, we conducted a RT-PCR ana­lysis targeting the virus sequences. Total nucleic acids were individually extracted from the cells of each isolate with SDS-phenol and used as the template. Two specific primer pairs were used in the RT-PCR ana­lysis, and the products were applied to direct Sanger sequencing.

The phylogenetic positions of the identified RNA viruses were elucidated based on a maximum likelihood-based phylogenetic tree using the deduced amino acid sequences of the RNA-dependent RNA polymerase (RdRp) gene. Details on this method and the accession numbers of the sequences used are shown in the supplemental material.

In RNA virus screening, 30 strains were pooled into pool-1 and pool-2, as shown in Table S1. The FLDS ana­lysis provided 45 and 30 contigs (>500 nt and >0.05% read abundance) from pool-1 and pool-2, respectively. These contigs were examined by BLASTX and HMM ana­lyses, and a single RNA virus contig was identified in pool-2. In the BLASTX ana­lysis, this viral contig showed the lowest e-value with RdRp of Bremia lactucae associated narnavirus 2 (BlaNV2) (e-value; 6E–57, identity 30%), a member of the family *Narnaviridae*. To identify the host of this narnavirus from pool-2, RT-PCR was performed with two sets of specific primers named Narna-P1 and Narna-P2 (Fig. 1A). PCR products were obtained only from strain YPF1522 (*Haloplacidia* sp. deposited in the National Institute for Environmental Studies collection as NIES-4585) (Fig. 1B), and sequences were identical to the narnavirus contig sequence (data not shown). Based on the host species and sequence similarity, we named this novel virus Haloplacidia narnavirus 1 (HpNV1). Although the culture of YPF1522 contained prey bacterial cells, the host of HpNV1 appeared to be *Haloplacidia* sp., not the prey bacteria, because all known hosts of narnaviruses are eukaryotes.

To obtain the complete genome sequence of HpNV1, a FLDS ana­lysis of strain YPF1522 was performed. The results obtained for HpNV1 revealed a bisegmented genome consisting of RNA1 and RNA2 (Fig. 1A) (GenBank accession: LC728461 and LC730475). These two full-length sequences shared both terminal sequences (Fig. 1C). Conserved terminal sequences are a hallmark of the genomic segment in a single RNA virus (Hutchinson *et al.*, 2010). The predicted amino acid sequence of the open reading frame (ORF) of RNA1 showed the lowest e-value with RdRp of BlaNV2, as described above. However, the ORFs of RNA2 did not show significant (e-value >1×10^–5^) similarities to known protein sequences.

To identify the phylogenetic position of HpNV1, we constructed a phylogenetic tree using the RdRp amino acid sequence of HpNV1 and viruses in the family *Narnaviridae* (Fig. 2). *Narnaviridae* currently includes one genus (*Narnavirus*), and only two viruses (*Saccharomyces 20S RNA narnavirus* and *Saccharomyces 23S RNA narnavirus*) are defined as species in this genus by the International Committee on Taxonomy of Viruses (https://talk.ictvonline.org/). The phylogenetic tree revealed that HpNV1 was distantly related to the genus *Narnavirus*. However, HpNV1 clustered together with known viruses in the family *Narnaviridae* detected in isolates of Trypanosomatid (Euglenozoa), Peronosporomycetes (Stramenopiles), and *Plasmodium* (Alveolata) and in environmental samples of invertebrates, fungi, and Peronosporomycetes. This result suggests that HpNV1 is a new member of an undefined genus in the family *Narnaviridae*.

RNA virus screening from various protist species was performed in the present study, and an RNA virus was detected from only one sample. Not all strains of the same species have RNA viruses, and the prevalence of RNA viruses differ among host species (Chiba *et al.*, 2020a, 2021). This might be the cause of the low frequency of the virus-infected samples in this study.

*Haloplacidia* sp. YPF1522 is a heterotrophic marine protist belonging to Stramenopiles (Rybarski *et al.*, 2021). Stramenopiles is a major eukaryotic group rooted between Bigyra and Gyrista (Adl *et al.*, 2019). Although narnaviruses have been identified in two groups of Gyrista (Ochrophyta and Peronosporomycetes) (Cai *et al.*, 2012; Charon *et al.*, 2021), none have been reported in Bigyra, which includes *Haloplacidia* (Rybarski *et al.*, 2021). Therefore, although we cannot rule out the possibility that the host of HpNV1 is prey bacteria, this is the first study of a narnavirus potentially infecting Bigyra. This result suggests that narnaviruses are more widely distributed in Stramenopiles than previously reported (Cai *et al.*, 2012; Charon *et al.*, 2021). In addition to previous findings (Charon *et al.*, 2021), the present results support the ubiquity of *Narnaviridae* in eukaryotes and the evolutionary hypothesis of the phylum *Lenarviricota* including the families *Narnaviridae*, *Leviviridae*, and *Mitoviridae* (Dolja and Koonin, 2018). *Narnaviridae* and *Mitoviridae* are eukaryotic RNA viruses that are proposed to originate from RNA bacteriophages in *Leviviridae*. The RNA bacteriophage that infected the ancestors of mitochondria was assumed to be brought into a eukaryotic ancestor during eukaryogenesis, and this is the ancestor of *Mitoviridae*. *Narnaviridae* is proposed to originate from mitoviruses that escaped to the cytosol. This scenario implies the ubiquity of *Narnaviridae* and *Mitoviridae* in eukaryotes.

Although the typical genomic organization of viruses in the family *Narnaviridae* is mono-segmented (King *et al.*, 2012), recent studies suggest that some narnaviruses have a bisegmented genome (Grybchuk *et al.*, 2018; Chiba *et al.*, 2020a; Jia *et al.*, 2021). Among them, Aspergillus lentulus narnavirus 1 (AleNV1) and Leptomonas seymouri narna‑like virus 1 (LepseyNLV1) clustered together with HpNV1 (Fig. 2), and their genome organizations are consistent with that of HpNV1 (Fig. S2). Therefore, although the relative dsRNA abundance of RNA1 and RNA2 differed (Table S2), we concluded that RNA2 is a genomic segment of HpNV1.

Narnaviruses lack an extracellular phase in their life cycle and persistently infect their hosts without lysis (King *et al.*, 2012). Viruses with this type of life cycle are called persistent-type viruses (Márquez and Roossinck, 2012; Urayama *et al.*, 2022). They are detected in various environmental samples and may be widely distributed in the RNA viral sequence space (Urayama *et al.*, 2022). However, the hosts of the majority of persistent-type viruses in the RNA virus sequence space have not yet been identified. Since the previously reported AuRNAV is an acute-type RNA virus that lyses host cells and enters into new cells, the present study is the first to report a persistent-type virus in Bigyra.

## Citation

Chiba, Y., Yabuki, A., Takaki, Y., Nunoura, T., Urayama, S., and Hagiwara, D. (2023) The First Identification of a Narnavirus in Bigyra, a Marine Protist. *Microbes Environ ***38**: ME22077.

https://doi.org/10.1264/jsme2.ME22077

## Supplementary Material

Supplementary Material

## Figures and Tables

**Fig. 1.**
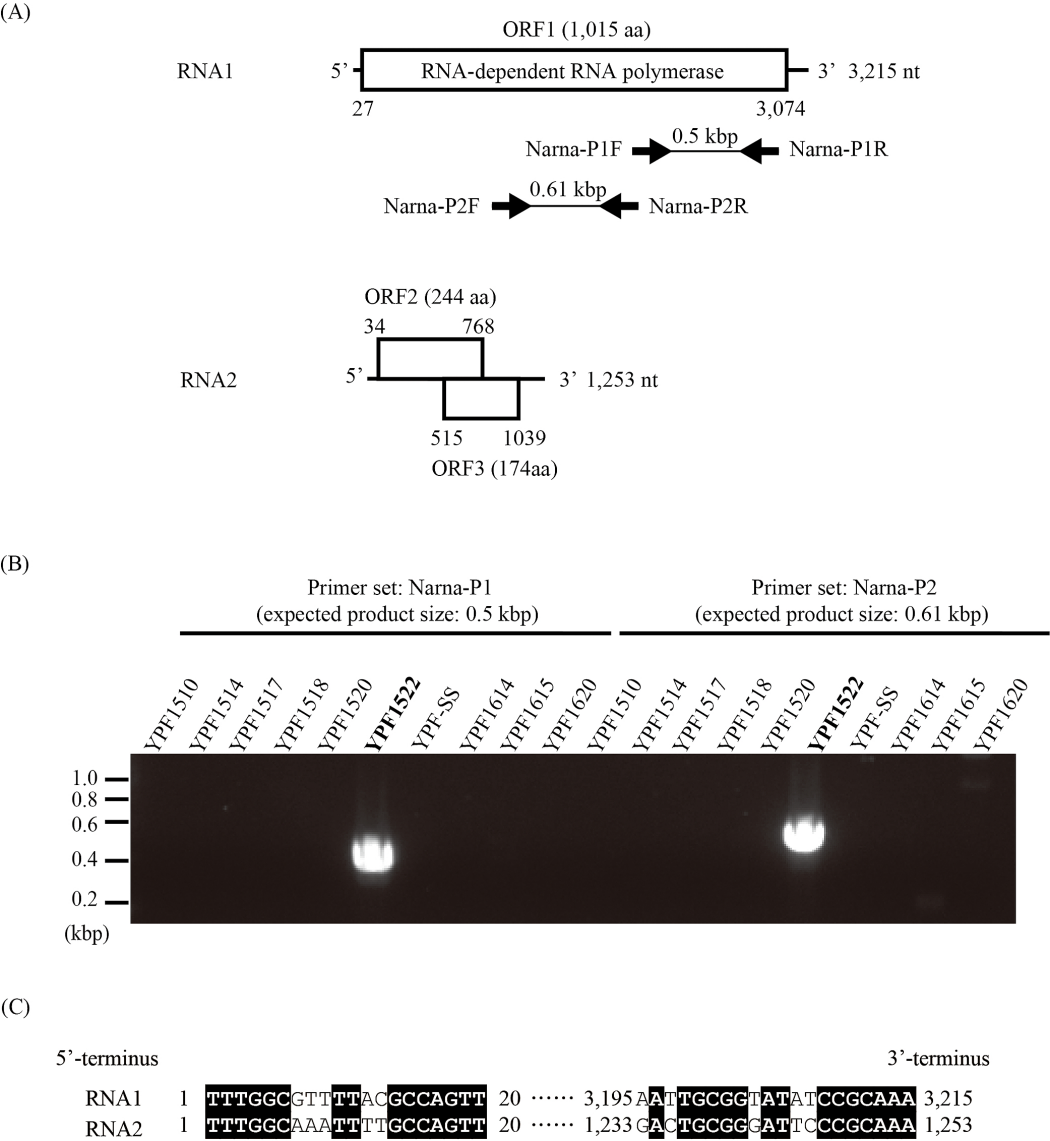
Host identification and genomic organization of HpNV. (A) Schematic representation of the HpNV1 genome and the position of the two primer pairs used in the RT-PCR ana­lysis. The boxes indicate open reading frames encoding >150 amino acid residues, and empty boxes show hypothetical proteins. The black arrows indicate the direction and location of each primer. (B) RT-PCR-based host identification of HpNV1 from pool-2. (C) Multiple alignments of the nucleotide sequences of both terminal regions in the coding strands of RNA1 and RNA2 of HpNV1. Black shading indicates nucleotide positions that are identical. Numbers represent nucleotide positions.

**Fig. 2.**
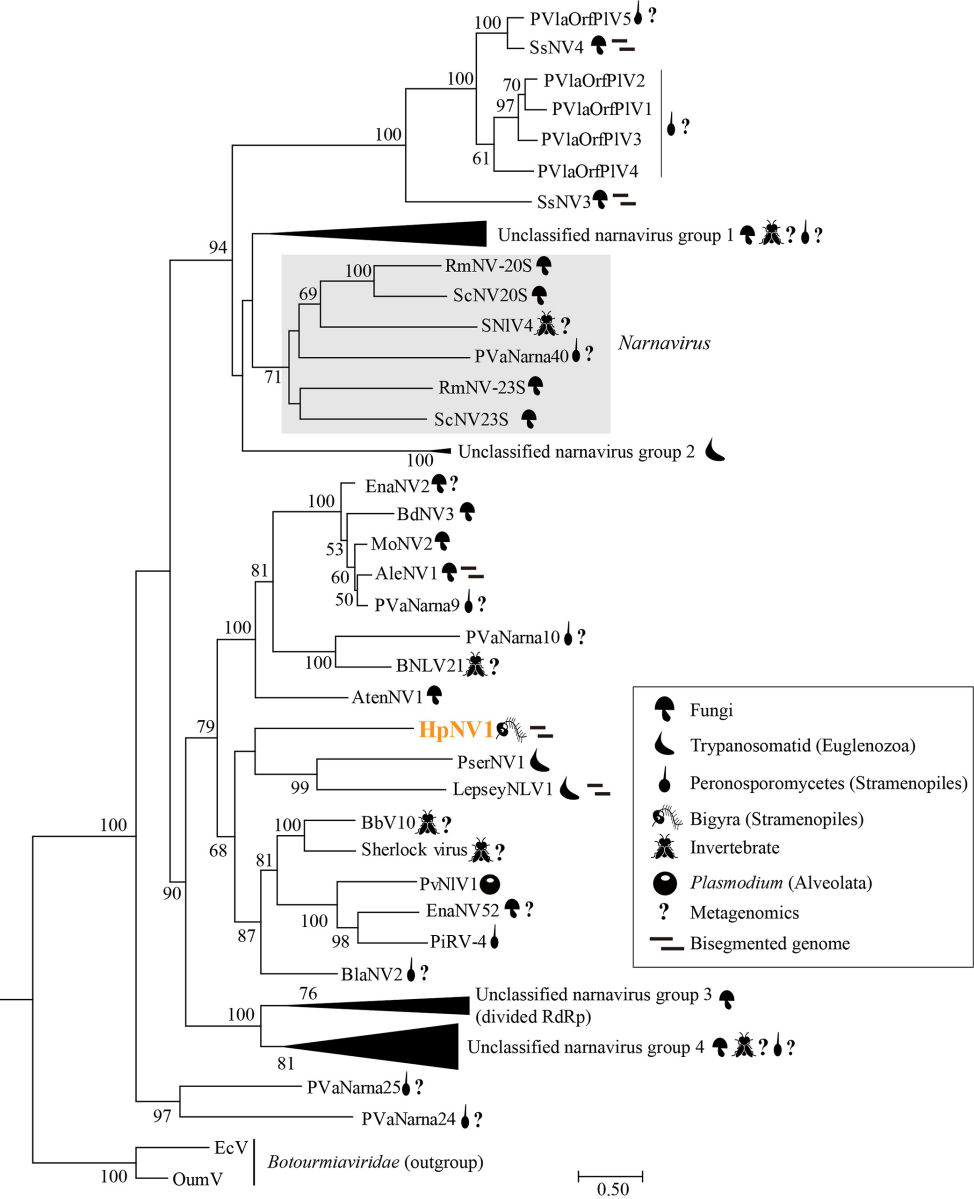
Phylogenetic tree for RdRp from the family *Narnaviridae*. Branch labels indicate bootstrap support (%) from 1,000 RAxML bootstrap samplings, and we showed more than 50% bootstrap support. Host taxa are shown by symbols. The scale bar indicates the number of amino acid substitutions per site. The best-fitting amino acid substitution model was [rtREV+F+G]. Full virus names and accession numbers are listed in Table S3. Viruses included in each collapsed node are indicated in Table S3. The newly described virus is shown in orange.

## References

[B1] Adl, S.M., Bass, D., Lane, C.E., Lukeš, J., Schoch, C.L., Smirnov, A., et al. (2019) Revisions to the Classification, Nomenclature, and Diversity of Eukaryotes. J Eukaryot Microbiol 66: 4–119. 30257078 10.1111/jeu.12691 PMC6492006

[B2] Bigot, T., Temmam, S., Pérot, P., and Eloit, M. (2019) RVDB-prot, a reference viral protein database and its HMM profiles. F1000Research 8: 530. 32983411 10.12688/f1000research.18776.1 PMC7492780

[B3] Brown, A.R., Lilley, M., Shutler, J., Lowe, C., Artioli, Y., Torres, R., et al. (2019) Assessing risks and mitigating impacts of harmful algal blooms on mariculture and marine fisheries. Rev Aquac 12: 1663–1688.

[B4] Burki, F., Roger, A.J., Brown, M.W., and Simpson, A.G.B. (2020) The new tree of eukaryotes. Trends Ecol Evol 35: 43–55. 31606140 10.1016/j.tree.2019.08.008

[B5] Cai, G., Myers, K., Fry, W.E., and Hillman, B.I. (2012) A member of the virus family Narnaviridae from the plant pathogenic oomycete Phytophthora infestans. Arch Virol 157: 165–169. 21971871 10.1007/s00705-011-1126-5

[B6] Charon, J., Grigg, M.J., Eden, J.-S., Piera, K.A., Rana, H., William, T., et al. (2019) Novel RNA viruses associated with Plasmodium vivax in human malaria and Leucocytozoon parasites in avian disease. PLoS Pathog 15: e1008216. 31887217 10.1371/journal.ppat.1008216 PMC6953888

[B7] Charon, J., Murray, S., and Holmes, E.C. (2021) Revealing RNA virus diversity and evolution in unicellular algae transcriptomes. Virus Evol 7: veab070. 36819971 10.1093/ve/veab070 PMC9927876

[B8] Chen, Y.-M., Sadiq, S., Tian, J.-H., Chen, X., Lin, X.-D., Shen, J.-J., et al. (2022) RNA viromes from terrestrial sites across China expand environmental viral diversity. Nat Microbiol 7: 1312–1323. 35902778 10.1038/s41564-022-01180-2

[B9] Chiba, Y., Oiki, S., Yaguchi, T., Urayama, S., and Hagiwara, D. (2020a) Discovery of divided RdRp sequences and a hitherto unknown genomic complexity in fungal viruses. Virus Evol 7: veaa101. 33505709 10.1093/ve/veaa101 PMC7816673

[B10] Chiba, Y., Tomaru, Y., Shimabukuro, H., Kimura, K., Hirai, M., Takaki, Y., et al. (2020b) Viral RNA genomes identified from marine macroalgae and a diatom. Microbes Environ 35: ME20016. 32554943 10.1264/jsme2.ME20016 PMC7511793

[B11] Chiba, Y., Oiki, S., Zhao, Y., Nagano, Y., Urayama, S., and Hagiwara, D. (2021) Splitting of RNA-dependent RNA polymerase is common in Narnaviridae: Identification of a type II divided RdRp from deep-sea fungal isolates. Virus Evol 7: veab095. 10.1093/ve/veab095 PMC10131275 37124704

[B12] Cobbin, J.C., Charon, J., Harvey, E., Holmes, E.C., and Mahar, J.E. (2021) Current challenges to virus discovery by meta-transcriptomics. Curr Opin Virol 51: 48–55. 34592710 10.1016/j.coviro.2021.09.007

[B13] Derevnina, L., Petre, B., Kellner, R., Dagdas, Y.F., Sarowar, M.N., Giannakopoulou, A., et al. (2016) Emerging oomycete threats to plants and animals. Philos Trans R Soc Lond B Biol Sci 371: 20150459. 28080985 10.1098/rstb.2015.0459 PMC5095538

[B14] Dolja, V.V., and Koonin, E.V. (2018) Metagenomics reshapes the concepts of RNA virus evolution by revealing extensive horizontal virus transfer. Virus Res 244: 36–52. 29103997 10.1016/j.virusres.2017.10.020 PMC5801114

[B15] Field, C.B., Behrenfeld, M.J., Randerson, J.T., and Falkowski, P. (1998) Primary production of the biosphere: integrating terrestrial and oceanic components. Science 281: 237–240. 9657713 10.1126/science.281.5374.237

[B16] Gilbert, K.B., Holcomb, E.E., Allscheid, R.L., and Carrington, J.C. (2019) Hiding in plain sight: New virus genomes discovered via a systematic ana­lysis of fungal public transcriptomes. PLoS One 14: e0219207. 31339899 10.1371/journal.pone.0219207 PMC6655640

[B17] Grybchuk, D., Akopyants, N.S., Kostygov, A.Y., Konovalovas, A., Lye, L.-F., Dobson, D.E., et al. (2018) Viral discovery and diversity in trypanosomatid protozoa with a focus on relatives of the human parasite Leishmania. Proc Natl Acad Sci U S A 115: E506–E515. 29284754 10.1073/pnas.1717806115 PMC5776999

[B18] Hirai, M., Takaki, Y., Kondo, F., Horie, M., Urayama, S., and Nunoura, T. (2021) RNA viral metagenome ana­lysis of subnanogram dsRNA using fragmented and primer ligated dsRNA sequencing (FLDS). Microbes Environ 36: ME20152. 33952860 10.1264/jsme2.ME20152 PMC8209451

[B19] Hutchinson, E.C., von Kirchbach, J.C., Gog, J.R., and Digard, P. (2010) Genome packaging in influenza A virus. J Gen Virol 91: 313–328. 19955561 10.1099/vir.0.017608-0

[B20] Jia, J., Fu, Y., Jiang, D., Mu, F., Cheng, J., Lin, Y., et al. (2021) Interannual dynamics, diversity and evolution of the virome in Sclerotinia sclerotiorum from a single crop field. Virus Evol 7: veab032. 33927888 10.1093/ve/veab032 PMC8058396

[B21] Kawasaki, J., Kojima, S., Tomonaga, K., and Horie, M. (2021) Hidden viral sequences in public sequencing data and warning for future emerging diseases. mBio 12: e01638-21. 34399612 10.1128/mBio.01638-21 PMC8406186

[B22] King, A.M.Q., Adams, M.J., Carstens, E.B., and Lefkowitz, E.J. (2012) *Virus Taxonomy: Classification and Nomenclature of Viruses: Ninth Report of the International Committee on Taxonomy of Viruses*. Amsterdam: Elsevier.

[B23] Márquez, L.M., and Roossinck, M.J. (2012) Do persistent RNA viruses fit the trade-off hypothesis of virulence evolution? Curr Opin Virol 2: 556–560. 22819020 10.1016/j.coviro.2012.06.010

[B24] Mifsud, J.C.O., Gallagher, R.V., Holmes, E.C., and Geoghegan, J.L. (2022) Transcriptome mining expands knowledge of RNA viruses across the plant kingdom. J Virol e0026022. 35638822 10.1128/jvi.00260-22 PMC9769393

[B25] Milne, I., Bayer, M., Cardle, L., Shaw, P., Stephen, G., Wright, F., and Marshall, D. (2010) Tablet--next generation sequence assembly visualization. Bioinformatics 26: 401–402. 19965881 10.1093/bioinformatics/btp666 PMC2815658

[B26] Mitra, A.K., and Mawson, A.R. (2017) Neglected tropical diseases: epidemiology and global burden. Trop Med Infect Dis 2: 36. 30270893 10.3390/tropicalmed2030036 PMC6082091

[B27] Morris, T.J. (1979) Isolation and ana­lysis of double-stranded RNA from virus-infected plant and fungal tissue. Phytopathology 69: 854. 10.1094/Phyto-69-854 40934407

[B28] Nagasaki, K., and Yamaguchi, M. (1997) Isolation of a virus infectious to the harmful bloom causing microalga Heterosigma akashiwo (Raphidophyceae). Aquat Microb Ecol 13: 135–140.

[B29] Neri, U., Wolf, Y.I., Roux, S., Camargo, A.P., Lee, B., Kazlauskas, D., et al. (2022) Expansion of the global RNA virome reveals diverse clades of bacteriophages. Cell 185: 4023–4037. 36174579 10.1016/j.cell.2022.08.023

[B30] Raghukumar, S., Ramaiah, N., and Raghukumar, C. (2001) Dynamics of thraustochytrid protists in the water column of the Arabian Sea. Aquat Microb Ecol 24: 175–186.

[B31] Rybarski, A.E., Nitsche, F., Soo Park, J., Filz, P., Schmidt, P., Kondo, R., et al. (2021) Revision of the phylogeny of Placididea (Stramenopiles): Molecular and morphological diversity of novel placidid protists from extreme aquatic environments. Eur J Protistol 81: 125809. 34673437 10.1016/j.ejop.2021.125809

[B32] Sakaguchi, S., Urayama, S., Takaki, Y., Hirosuna, K., Wu, H., Suzuki, Y., et al. (2022) NeoRdRp: A comprehensive dataset for identifying RNA-dependent RNA polymerases of various RNA viruses from metatranscriptomic data. Microbes Environ 37: ME22001. 36002304 10.1264/jsme2.ME22001 PMC9530720

[B33] Shi, M., Lin, X.-D., Tian, J.-H., Chen, L.-J., Chen, X., Li, C.-X., et al. (2016) Redefining the invertebrate RNA virosphere. Nature 540: 539–543. 27880757 10.1038/nature20167

[B34] Shi, M., Lin, X.-D., Chen, X., Tian, J.-H., Chen, L.-J., Li, K., et al. (2018) The evolutionary history of vertebrate RNA viruses. Nature 556: 197–202. 29618816 10.1038/s41586-018-0012-7

[B35] Starr, E.P., Nuccio, E.E., Pett-Ridge, J., Banfield, J.F., and Firestone, M.K. (2019) Metatranscriptomic reconstruction reveals RNA viruses with the potential to shape carbon cycling in soil. Proc Natl Acad Sci U S A 116: 25900–25908. 31772013 10.1073/pnas.1908291116 PMC6926006

[B36] Sutela, S., Forgia, M., Vainio, E.J., Chiapello, M., Daghino, S., Vallino, M., et al. (2020) The virome from a collection of endomycorrhizal fungi reveals new viral taxa with unprecedented genome organization. Virus Evol 6: veaa076. 33324490 10.1093/ve/veaa076 PMC7724248

[B37] Takao, Y., Nagasaki, K., Mise, K., Okuno, T., and Honda, D. (2005) Isolation and characterization of a novel single-stranded RNA Virus infectious to a marine fungoid protist, Schizochytrium sp. (Thraustochytriaceae, Labyrinthulea). Appl Environ Microbiol 71: 4516–4522. 16085844 10.1128/AEM.71.8.4516-4522.2005 PMC1183295

[B38] Tashyreva, D., Prokopchuk, G., Votýpka, J., Yabuki, A., Horák, A., and Lukeš, J. (2018) Life cycle, ultrastructure, and phylogeny of new diplonemids and their endosymbiotic bacteria. mBio 9: e02447–17. 29511084 10.1128/mBio.02447-17 PMC5845003

[B39] Urayama, S., Takaki, Y., and Nunoura, T. (2016) FLDS: A comprehensive dsRNA sequencing method for intracellular RNA virus surveillance. Microbes Environ 31: 33–40. 26877136 10.1264/jsme2.ME15171 PMC4791113

[B40] Urayama, S., Takaki, Y., Nishi, S., Yoshida-Takashima, Y., Deguchi, S., Takai, K., and Nunoura, T. (2018) Unveiling the RNA virosphere associated with marine microorganisms. Mol Ecol Resour 18: 1444–1455. 30256532 10.1111/1755-0998.12936

[B41] Urayama, S., Takaki, Y., Chiba, Y., Zhao, Y., Kuroki, M., Hagiwara, D., and Nunoura, T. (2022) Eukaryotic microbial RNA viruses-acute or persistent? Insights into their function in the aquatic ecosystem. Microbes Environ 37: ME22034. 35922920 10.1264/jsme2.ME22034 PMC9763035

[B42] Wolf, Y.I., Kazlauskas, D., Iranzo, J., Lucía-Sanz, A., Kuhn, J.H., Krupovic, M., et al. (2018) Origins and evolution of the global RNA virome. mBio 9: e02329-18. 30482837 10.1128/mBio.02329-18 PMC6282212

[B43] Wolf, Y.I., Silas, S., Wang, Y., Wu, S., Bocek, M., Kazlauskas, D., et al. (2020) Doubling of the known set of RNA viruses by metagenomic ana­lysis of an aquatic virome. Nat Microbiol 5: 1262–1270. 32690954 10.1038/s41564-020-0755-4 PMC7508674

[B44] World Health Organization. (2019) World Malaria Report 2018. Geneva: WHO.

[B45] Yabuki, A., and Tame, A. (2015) Phylogeny and Reclassification of Hemistasia phaeocysticola (Scherffel) Elbrächter & Schnepf, 1996. J Eukaryot Microbiol 62: 426–429. 25377132 10.1111/jeu.12191

[B46] Zayed, A.A., Wainaina, J.M., Dominguez-Huerta, G., Pelletier, E., Guo, J., Mohssen, M., et al. (2022) Cryptic and abundant marine viruses at the evolutionary origins of Earth’s RNA virome. Science 376: 156–162. 35389782 10.1126/science.abm5847 PMC10990476

