# Supplementary material for: The First Identification of a Narnavirus in Bigyra, a Marine Protist
Source: Microbes Environ. 2023 Mar 1;38(1):ME22077. doi: 10.1264/jsme2.ME22077 (PMC10037099; doi:10.1264/jsme2.ME22077)
Supplement: Supplementary file 1 — Supplementary Material [file 38_22077_s1.pdf]

Supplemental material

Details of materials and methods

Culture conditions

Thirty protists were cultured with Hemi medium (Tashyreva *et al.*, 2018) or KLB medium (Yabuki and Tame, 2015) at 20°C. The taxonomic information of each protist is summarized in Table S1.

Nucleic acid extraction and dsRNA-sequencing from pooled samples

The cells were harvested from cultures by centrifugation at 2,400 × *g* for 4 min. Cells of these strains were pooled into pool-1 and -2 as shown in Table S1. The purification and sequencing of dsRNA was performed as described previously (Urayama *et al.*, 2018). In brief, cells were disrupted in liquid nitrogen using a mortar. Total nucleic acid was extracted from ground cells with SDS-phenol. dsRNA was purified from the total nucleic acids by cellulose resin. The remaining DNA and single-stranded RNA were digested by amplification grade DNase I (Invitrogen, Carlsbad, CA, USA) and S1 nuclease (Invitrogen).

The purified dsRNA was converted into double-stranded cDNA (dscDNA) by FLDS ver. 2 (Urayama *et al.*, 2018). In brief, the dsRNA was fragmented by ultrasonication using a Covaris S220 ultrasonicator (Woburn, MA, USA). The U2 primer was ligated to the fragmented dsRNA using T4 RNA ligase (Takara Bio Inc., Kusatsu, Japan), and 1st-strand cDNA was synthesized by a SMARTer RACE 5′/3′ Kit (Takara Bio) with the U2-complementary primer. After PCR amplification, we constructed Illumina sequencing libraries using the KAPA Hyper Prep Kit Illumina platform (Kapa Biosystems). The libraries were sequenced by an Illumina NovaSeaq 6000 platform with 150 bp paired-end sequences (Illumina, CA, USA). More than one million reads were obtained for each library.

Data processing

To obtain cleaned reads, we removed low-quality, adapter, low-complexity, and rRNA sequences from the raw sequence reads as described previously (Hirai *et al.*, 2021) with a custom Perl script (https://github. com/takakiy/FLDS). Following previous reports (Urayama *et al.*, 2016, 2018), each cleaned read was assembled *de novo* using CLC GENOMICS WORKBENCH version 11.0 (CLC Bio, Aarhus, Denmark). To obtain full-length sequences, assembled contigs were manually extended. To extend the contig sequence, re-mapping the contigs were conducted using CLC Genomics Workbench version 11.0, and the results were visualized using a tablet viewer (Milne *et al.* 2010). Then, partially aligned reads at the terminus of contigs were manually collected by viewing the alignment and re-assembled. These operations were repeated until the extension of the contig was finished. If 10 or more reads stopped in the same position around the end of the contig, we recognized the position as the terminal end. When both ends of the contig were defined as termini, the contig was defined as a full-length sequence. The assembled contigs were annotated by BLASTX analysis against the NCBI non-redundant protein database and RNA viral protein sequences detected in recent RNA virome studies (Chen *et al.*, 2022; Neri *et al.*, 2022; Zayed *et al.*, 2022). To identify more distantly related RNA viruses, we performed RNA virus detection based on hidden Markov model (HMM) profiles. Contigs without any BLASTX hits at the 1×10^{-5} e-value cutoff were submitted against RVDB-prot (Bigot *et al.*, 2019) and NeoRdRp (Sakaguchi *et al.*, 2022) using the HMMer3 program with the default parameters and e-value 1×10^{-5} threshold (Eddy, 2011).

RT-PCR analysis

To identify the host organism of the RNA virus detected in pooled sequence data, we conducted RT-PCR analyses targeting the virus sequence. RT-PCR analysis was performed on each isolate contained in poo1-2. Total nucleic acids were individually extracted from cells of each isolate with SDS-phenol and were used as the template. In this RT-PCR analysis, we used two specific primer pairs: Narna-P1F (5'- GGT ACG AAA AGG CCC GAT CA-3') and Narna-P1R (5'- ACA AGG CTC ATC TCC GCA AA -3'); and Narna-P2F (5'- TCG TCT TGG TCT TGA GCG TC-3') and

Narna-P2R (5'- ATA CGC CCT CTT TGG AAC GG -3'). RT-PCR was performed using the SuperScript III One-Step RT-PCR System with Platinum Taq (Invitrogen) according to the manufacturer's protocol. PCR products were confirmed on a 1% agarose gel. Nucleic acids were stained with Gel Red (Biotium, CA, USA). The amplified fragments were excised and purified using a FastGene Gel/PCR Extraction Kit (Nippon Genetics, Tokyo, Japan). The products then were applied for direct Sanger sequencing.

DsRNA-sequencing from YPF1522

To obtain the complete genome sequence of the detected virus, we constructed a dsRNA sequencing library from cells of YPF1522. The cells were collected from cultures by centrifugation at 2,400 × *g* for 4 min, and dsRNA was purified from 0.16 g of the collected cells as described above. The purified dsRNA was converted into dscDNA by FLDS ver. 3 (Hirai *et al.*, 2021). The resultant dscDNA was used for the construction of the Illumina sequencing library as mentioned above, and the library was sequenced using the Illumina MiSeq platform with 300 bp paired-end sequences (Illumina). More than 400,000 reads were obtained for each library. The raw sequence reads were processed as mentioned above (see Data processing).

Phylogenetic analysis

In the phylogenetic analysis, the deduced amino acid sequences of the putative RdRp domains and related sequences were aligned using MUSCLE (Edgar, 2004) in MEGA6 (Tamura *et al.*, 2013). The ambiguous positions in the alignment were removed with trimAl (option: -gt 0.9) (Capella-Gutiérrez *et al.*, 2009). A maximum likelihood-based phylogenetic tree was constructed using RAxML (Stamatakis, 2014). The amino acid substitution model was selected by Aminosan (Tanabe, 2011) using corrected Akaike's information criterion (Sugiura, 1978). MEGA6 was used for visualization of phylogenetic trees.

References

Bigot, T., Temmam, S., Pérot, P., and Eloit, M. (2019) RVDB-prot, a reference viral protein database and its HMM profiles. *F1000Res* **8**: 530.

Capella-Gutiérrez, S., Silla-Martínez, J.M., and Gabaldón, T. (2009) trimAl: a tool for automated alignment trimming in large-scale phylogenetic analyses. *Bioinformatics* **25**: 1972–1973.

Chen, Y.-M., Sadiq, S., Tian, J.-H., Chen, X., Lin, X.-D., Shen, J.-J., *et al.* (2022) RNA viromes from terrestrial sites across China expand environmental viral diversity. *Nat Microbiol* **7**: 1312–1323.

Eddy, S.R. (2011) Accelerated Profile HMM Searches. *PLoS Comput Biol* **7**: e1002195.

Edgar, R.C. (2004) MUSCLE: multiple sequence alignment with high accuracy and high throughput. *Nucleic Acids Res* **32**: 1792–1797.

Hirai, M., Takaki, Y., Kondo, F., Horie, M., Urayama, S.-I., and Nunoura, T. (2021) RNA Viral Metagenome Analysis of Subnanogram dsRNA Using Fragmented and Primer Ligated dsRNA Sequencing (FLDS). *Microbes Environ* **36**: ME20152..

Milne, I., Bayer, M., Cardle, L., Shaw, P., Stephen, G., Wright, F., and Marshall, D. (2010) Tablet--next generation sequence assembly visualization. *Bioinformatics* **26**: 401–402.

Neri, U., Wolf, Y.I., Roux, S., Camargo, A.P., Lee, B., Kazlauskas, D., *et al.* (2022) Expansion of the global RNA virome reveals diverse clades of bacteriophages. *Cell* ***185**: 4023–4037.*

Sakaguchi, S., Urayama, S., Takaki, Y., Hirosuna, K., Wu, H., Suzuki, Y., *et al.* (2022) NeoRdRp: A Comprehensive Dataset for Identifying RNA-dependent RNA Polymerases of Various RNA Viruses from Metatranscriptomic Data. *Microbes Environ* **37**: ME22001.

Stamatakis, A. (2014) RAxML version 8: a tool for phylogenetic analysis and post-analysis of large phylogenies. *Bioinformatics* **30**: 1312–1313.

Sugiura, N. (1978) Further analysts of the data by akaike' s information criterion and the finite corrections. *Communications in Statistics - Theory and Methods* **7**: 13–26.

Tamura, K., Stecher, G., Peterson, D., Filipski, A., and Kumar, S. (2013) MEGA6: Molecular Evolutionary Genetics Analysis version 6.0. *Mol Biol Evol* **30**: 2725–2729.

Tanabe, A.S. (2011) Kakusan4 and Aminosan: two programs for comparing nonpartitioned,

proportional and separate models for combined molecular phylogenetic analyses of multilocus sequence data. *Mol Ecol Resour* **11**: 914–921.

Tashyreva, D., Prokopchuk, G., Votýpka, J., Yabuki, A., Horák, A., and Lukeš, J. (2018) Life Cycle, Ultrastructure, and Phylogeny of New Diplonemids and Their Endosymbiotic Bacteria. *MBio* **9**: e02447–17.

Urayama, S., Takaki, Y., and Nunoura, T. (2016) FLDS: A Comprehensive dsRNA Sequencing Method for Intracellular RNA Virus Surveillance. *Microbes Environ* **31**: 33–40.

Urayama, S., Takaki, Y., Nishi, S., Yoshida-Takashima, Y., Deguchi, S., Takai, K., and Nunoura, T. (2018) Unveiling the RNA virosphere associated with marine microorganisms. *Mol Ecol Resour* **18**: 1444–1455.

Yabuki, A. and Tame, A. (2015) Phylogeny and Reclassification of Hemistasia phaeocysticola (Scherffel) Elbrächter & Schnepf, 1996. *J Eukaryot Microbiol* **62**: 426–429.

Zayed, A.A., Wainaina, J.M., Dominguez-Huerta, G., Pelletier, E., Guo, J., Mohssen, M., *et al.* (2022) Cryptic and abundant marine viruses at the evolutionary origins of Earth's RNA virome. *Science* **376**: 156–162.

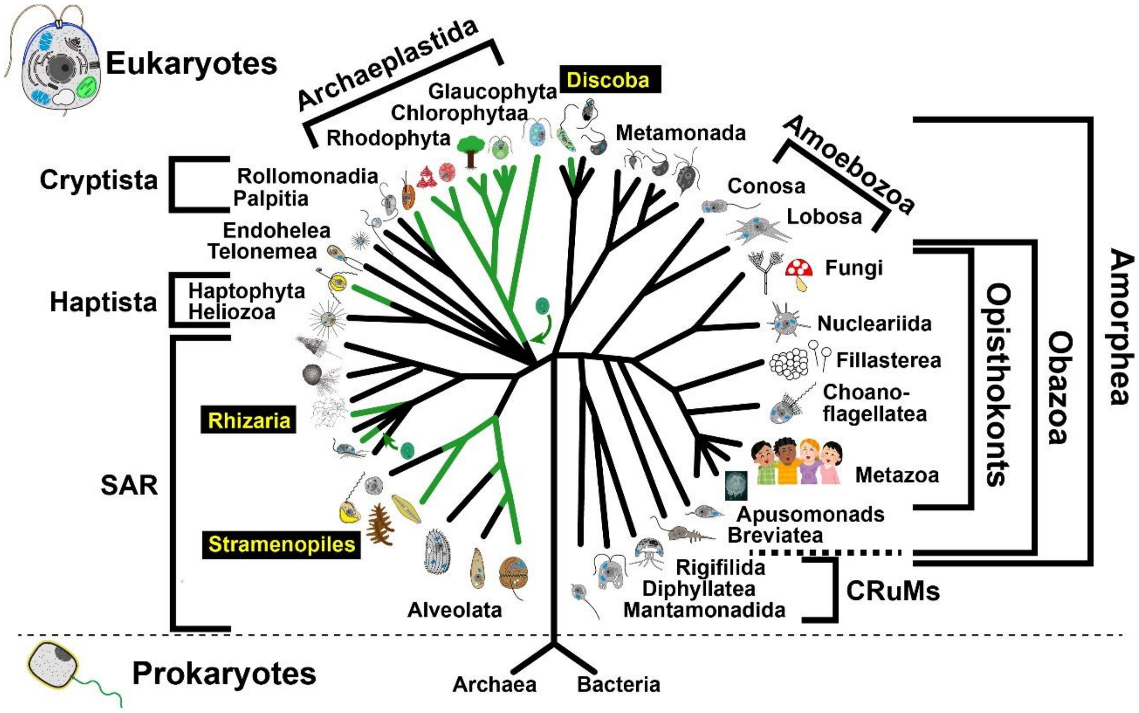

Fig. S1. Eukaryote cladogram based on Adl *et al*. (2019). The protists studied in this study belong to the groups shown by yellow latter in black box.

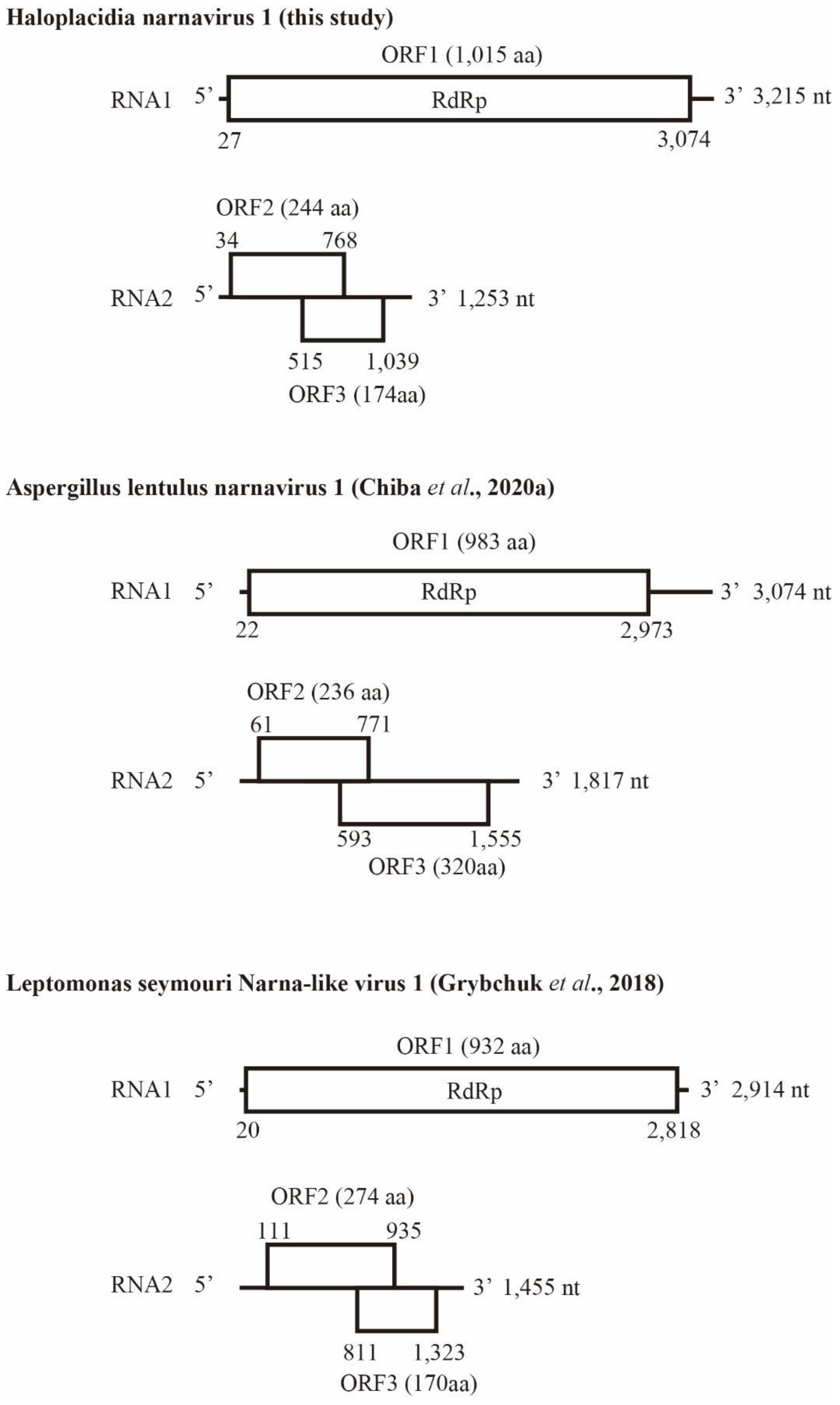

Fig. S2. Schematic representation of genomes of bisegmented narnaviruses. The boxes indicate open reading frames encoding >150 amino acid residues, and empty boxes show hypothetical proteins.

Table S1. List of isolates used in this study.

Pool	Isolate	Taxonomic identification*
Pool-1	YPF1514	Rhizaria, Cercozoa, Thecofilosea, Cryomonadida, Cryomonadida sp.
Pool-1	YPF1508	Discoba, Euglenozoa, Diplonemea, Diplonemidae, *Diplonema* sp.
Pool-1	YPF1513	Discoba, Euglenozoa, Diplonemea, Diplonemidae, *Rhynchopus* sp.
Pool-1	YPF1515	Discoba, Euglenozoa, Diplonemea, Diplonemidae, *Rhynchopus serpens*
Pool-1	YPF1516	Discoba, Euglenozoa, Diplonemea, Diplonemidae, *Rhynchopus* sp.
Pool-1	YPF1521	Discoba, Euglenozoa, Diplonemea, Diplonemidae, *Rhynchopus* sp.
Pool-1	YPF1601	Discoba, Euglenozoa, Diplonemea, Diplonemidae, *Lacrimia lanifica*
Pool-1	YPF1602	Discoba, Euglenozoa, Diplonemea, Diplonemidae, *Lacrimia* sp.
Pool-1	YPF1603	Discoba, Euglenozoa, Diplonemea, Diplonemidae, *Diplonema japonicum*
Pool-1	YPF1604	Discoba, Euglenozoa, Diplonemea, Diplonemidae, *Diplonema japonicum*
Pool-1	YPF1611	Discoba, Euglenozoa, Diplonemea, Diplonemidae, *Lacrimia* sp.
Pool-1	YPF1612	Discoba, Euglenozoa, Diplonemea, Diplonemidae, *Lacrimia* sp.
Pool-1	YPF1616	Discoba, Euglenozoa, Diplonemea, Diplonemidae, *Rhynchopus* sp.
Pool-1	YPF1617	Discoba, Euglenozoa, Diplonemea, Diplonemidae, *Rhynchopus* sp.
Pool-1	YPF1618	Discoba, Euglenozoa, Diplonemea, Diplonemidae, *Sulcionema specki*
Pool-1	YPF1621	Discoba, Euglenozoa, Diplonemea, Hemistasiidae, *Namystynia karyoxenos*
Pool-1	YPF1622	Discoba, Euglenozoa, Diplonemea, Hemistasiidae sp.
Pool-1	YPF1303 (NIES-3356)	Discoba, Euglenozoa, Diplonemea, Hemistasiidae, *Hemistasia paheocusticola*
Pool-1	YPF1605	Discoba, Euglenozoa, Diplonemea, Diplonemidae, *Diplonema aggregatum*
Pool-1	YPF1609	Discoba, Euglenozoa, Diplonemea, Hemistasiidae, *Hemistasia paheocusticola*
Pool-2	YPF1510	Stramenopiles, Bigyra, Sagenista, Labyrinthulomycetes, Labyrinthulida, *Aplanochytrium* sp.
Pool-2	YPF1517	Rhizaria, Cercozoa, Thecofilosea, Cryomonadida sp.
Pool-2	YPF1518	Rhizaria, Cercozoa, Thecofilosea, Cryomonadida sp.
Pool-2	YPF1522 (NIES-4585)	Stramenopiles, Bigyra, Opalozoa, Placidida, *Haloplacidia* sp.
Pool-2	YPF1514	Rhizaria, Cercozoa, Thecofilosea, Cryomonadida sp.
Pool-2	YPF-SS	Stramenopiles, Bigyra, Sagenista, Labyrinthulomycetes, Labyrinthulida ,*Aplanochytrium* sp.
Pool-2	YPF1614	Rhizaria, Cercozoa, Thecofilosea, Cryomonadida sp.
Pool-2	YPF1615	Rhizaria, Cercozoa, Thecofilosea, Cryomonadida sp.
Pool-2	YPF1620	Rhizaria, Cercozoa, Imbricatea, *Discomonas* sp.
Pool-2	YPF1520	Stramenopiles, Bigyra, Opalozoa, Bicosoecida, *Caecitellus* sp.

*Each strain was identified by careful microscopic observation and 18S rRNA gene sequence, and taxonomic assignments were basically in accordance with Adl *et al*. (2019).

Table S2. Read abundance of RNA1 and RNA2 of HpNV1.

	Length (bp)	Mapped read abundance (%)
RNA1	3,215	58.3
RNA2	1,253	0.9

Table S3. List of accession numbers and the full virus names used for sequence alignments and the phylogenetic analysis.

Abbreviation	Full virus name	Accession no.	UNG*
AleNV1	Aspergillus lentulus narnavirus 1	LC553702.1	0
BbV10	Beihai barnacle virus 10	NC_032428.1	0
BdNV3	Botryosphaeria dothidea narnavirus 3	MT103581.1	0
BlaNV2	Bremia lactucae associated narnavirus 2	MN565683.1	0
BNLV21	Beihai narna-like virus 21	NC_032406.1	0
EnaNV2	Erysiphe necator associated narnavirus 2	MN557020.1	0
EnaNV52	Erysiphe necator associated narnavirus 52	MN605465.1	0
LepseyNLV1	Leptomonas seymouri Narna-like virus 1	NC_040764	0
MoNV2	Magnaporthe oryzae narnavirus 2	LC553714.1	0
ONLV1	Ochlerotatus-associated narna-like virus 1	KF298275	0
ONLV2	Ochlerotatus-associated narna-like virus 2	KF298276	0
PiRV-4	Phytophthora infestans RNA virus 4	NC_029782	0
PserNV1	Phytomonas serpens narnavirus 1	NC_030308	0
PVaNarna9	Plasmopara viticola lesion associated narnavirus 9	MN539826	0
PVaNarna10	Plasmopara viticola lesion associated narnavirus 10	MN539827	0
PVaNarna24	Plasmopara viticola lesion associated narnavirus 24	MN539844	0
PVaNarna25	Plasmopara viticola lesion associated narnavirus 25	MN539845	0
PVaNarna40	Plasmopara viticola lesion associated narnavirus 40	MN539857	0
PVlaOrfPlV1	Plasmopara viticola lesion associated orfanplasmovirus 1	MT338024	0
PVlaOrfPlV2	Plasmopara viticola lesion associated orfanplasmovirus 2	MT338025	0
PVlaOrfPlV3	Plasmopara viticola lesion associated orfanplasmovirus 3	MT338026	0
PVlaOrfPlV4	Plasmopara viticola lesion associated orfanplasmovirus 4	MT338027	0
PVlaOrfPlV5	Plasmopara viticola lesion associated orfanplasmovirus 5	MT338028	0
PvNlV1	Plasmodium vivax Narna-Like virus 1	MN860568	0
RmNV-20S	Rhizopus microsporus 20S narnavirus	MK204624	0
RmNV-23S	Rhizopus microsporus 23S narnavirus	MK204625	0
ScNV20S	Saccharomyces 20S RNA narnavirus	NC_004051	0

ScNV23S	Saccharomyces 23S RNA narnavirus	NC_004050	0
Sherlock_virus	Sherlock virus	MN167477.1	0
SNlV4	Shahe narna-like virus 4	KX883556	0
SsNV3	Sclerotinia sclerotiorum narnavirus 3	MW442873	0
SsNV4	Sclerotinia sclerotiorum narnavirus 4	MW442875	0
AtenNV1	Aspergillus tennesseensis narnavirus 1	LC648937, LC648938	0
CvNV1	Coquillettidia venezuelensis narnavirus 1	MK285333	1
FpNV1	Fusarium poae narnavirus 1	NC_030865	1
PVaNarna15	Plasmopara viticola lesion associated narnavirus 15	MN539832	1
PVaNarna16	Plasmopara viticola lesion associated narnavirus 16	MN539833	1
PVaNarna17	Plasmopara viticola lesion associated narnavirus 17	MN539834	1
PVaNarna18	Plasmopara viticola lesion associated narnavirus 18	MN539835	1
PVaNarna42	Plasmopara viticola lesion associated narnavirus 42	MN539859	1
PVaNarna43	Plasmopara viticola lesion associated narnavirus 43	MN539860	1
BlunNV1	Blechmonas luni narnavirus 1	NC_040829	2
BwenNV1	Blechomonas wendygibsoni narnavirus 1	NC_040641	2
AfuNV2	Aspergillus fumigatus narnavirus 2	LC553688.1, LC553689.1	3
BcbNV1	Botrytis cinerea binarnavirus 1	MN619795.1, MT711186.1	3
BcbNV2	Botrytis cinerea binarnavirus 2	MN619796.1, MT119676.2	3
BcbNV3	Botrytis cinerea binarnavirus 3	MN619797.1, MT711185.1	3
BcbNV5	Botrytis cinerea binarnavirus 5	MN619799.1, MT711187.1	3
MoNV1	Magnaporthe oryzae narnavirus 1	LC553710.1, LC553711.1	3
OmSPV1	Oidiodendron maius splipalmivirus 1	MN736964, MN736965	3
AcreNV1	Aspergillus creber narnavirus 1	LC648933	4
BNlV22	Beihai narna-like virus 22	NC_032404	4
BNlV23	Beihai narna-like virus 23	NC_032457	4

NpNV1	Neofusicoccum parvum narnavirus 1	MK584833	4
PVaNarna11	Plasmopara viticola lesion associated narnavirus 11	MN539828	4
PVaNarna12	Plasmopara viticola lesion associated narnavirus 12	MN539829	4
PVaNarna13	Plasmopara viticola lesion associated narnavirus 13	MN539830	4
PVaNarna14	Plasmopara viticola lesion associated narnavirus 14	MN539831	4
PVaNarna26	Plasmopara viticola lesion associated narnavirus 26	MN539846	4
PVaNarna30	Plasmopara viticola lesion associated narnavirus 30	MN539847	4
PVaNarna31	Plasmopara viticola lesion associated narnavirus 31	MN539848	4
PVaNarna32	Plasmopara viticola lesion associated narnavirus 32	MN539849	4
PVaNarna33	Plasmopara viticola lesion associated narnavirus 33	MN539850	4
PVaNarna34	Plasmopara viticola lesion associated narnavirus 34	MN539851	4
PVaNarna35	Plasmopara viticola lesion associated narnavirus 35	MN539852	4
PVaNarna36	Plasmopara viticola lesion associated narnavirus 36	MN539853	4
PVaNarna37	Plasmopara viticola lesion associated narnavirus 37	MN539854	4
WiNV2	Wilkie narna-like virus 2	NC_035120	4
EcV	Epirus cherry virus	NC_011065.1	0
OumV	Ourmia melon virus	NC_011068.1	0

*UNG is the abbreviation of unclassified narnavirus groups (compressed nodes) shown in Figure 2. The number of UNG indicates which group the virus was included in. UNG 0 shows that the virus was not included in any unclassified narnavirus groups".
